# Cost implications of early treatment discontinuation in cancer: a real-world data analysis

**DOI:** 10.1093/oncolo/oyaf436

**Published:** 2026-02-25

**Authors:** H Colinda Post, Kirsten Opmeer, Tim Schutte, Marijke H Delsing, Lonneke Timmers, Carla E M Hollak, Hanneke W M van Laarhoven, Geert W J Frederix

**Affiliations:** Amsterdam UMC location University of Amsterdam, Department of Oncology, Amsterdam, The Netherlands; Cancer Center Amsterdam, Cancer Treatment and Quality of Life, Amsterdam, The Netherlands; Platform “Medicijn voor de Maatschappij” (Medicine for Society), Amsterdam, The Netherlands; Amsterdam UMC location Vrije Universiteit Amsterdam, Department of Oncology, Amsterdam, The Netherlands; Julius Center for Health Sciences and Primary Care, University Medical Center Utrecht, Utrecht, The Netherlands; Amsterdam UMC location Vrije Universiteit Amsterdam, Department of Oncology, Amsterdam, The Netherlands; The Netherlands Cancer Institute (NKI-AVL), Department of Medical Oncology, Amsterdam, The Netherlands; Scientific Advisory Board, National Health Care Institute (Zorginstituut Nederland), Diemen, The Netherlands; Scientific Advisory Board, National Health Care Institute (Zorginstituut Nederland), Diemen, The Netherlands; Erasmus School of Health, Policy and Management, Rotterdam, The Netherlands; Platform “Medicijn voor de Maatschappij” (Medicine for Society), Amsterdam, The Netherlands; Amsterdam UMC location University of Amsterdam, Department of Endocrinology and Metabolism, Amsterdam, The Netherlands; Amsterdam UMC location University of Amsterdam, Department of Oncology, Amsterdam, The Netherlands; Cancer Center Amsterdam, Cancer Treatment and Quality of Life, Amsterdam, The Netherlands; Amsterdam UMC location Vrije Universiteit Amsterdam, Department of Oncology, Amsterdam, The Netherlands; Julius Center for Health Sciences and Primary Care, University Medical Center Utrecht, Utrecht, The Netherlands

**Keywords:** cancer, systemic therapy, early treatment discontinuation, end-of-life care, healthcare costs

## Abstract

**Background:**

Health care costs are rising due to increasing cancer incidence and the expanding use of high-cost anticancer medicines. Early treatment discontinuation (ETD) may signal inefficiencies in medicine use or reflect appropriate or inevitable clinical decisions. Despite its clinical and economic relevance, national-level data on ETD remain limited. This study aims to quantify ETD rates and associated costs for the highest budget anticancer medicines in the Netherlands and assess trends from 2018 to 2022.

**Methods:**

We performed a retrospective analysis using real-world data from the Dutch national claims database. ETD was defined as treatment discontinued within 90 days. The study focused on the 30 highest-budget impact anticancer medicines in 2022, assessing ETD rates, related medicine costs, and trends over 5 years (2018-2022).

**Results:**

In 2022, these medicines accounted for €783 million in expenditures, with ETD representing 9.9% (€77 million). Among 30 343 treatments, 29.7% (9025) were discontinued within 90 days. From 2018 to 2022, total medication costs increased by 27.1%, while ETD costs rose by 9.6%. ETDs increased from 7287 to 9025 (+23.9%), with substantial variation among medicines. For most medicines, survivors accounted for most ETD spending, while ETD followed by death remained 9%.

**Conclusions:**

Approximately 30% of anticancer treatments are discontinued early, accounting for nearly 10% of medicine costs. While ETD highlights opportunities to improve efficiency, it also includes clinically justified and unavoidable discontinuations. Efforts to reduce avoidable ETD through improved patient selection, toxicity prediction, and treatment optimization are essential for more rational and equitable use of high-cost anticancer therapies.

Implications for Practice Nearly 30% of costly anticancer treatments in the Netherlands are discontinued within 90 days, representing about 10% (€77 million in 2022) of total medicine costs. This persistently high rate of early treatment discontinuation (ETD) may indicate inefficiencies in therapy use. To improve outcomes, reduce toxicities, and lower avoidable costs, targeted interventions and better patient selection are urgently needed. Reducing ETD can enhance patient quality of life and support clinicians and policymakers to deliver more efficient, rational, and cost-effective cancer care.

## Introduction

For decades, the demand for healthcare services has been rising, also in anticancer treatment and diagnosis, contributing to a rise in costs.^1^^,^^2^ This trend is driven by the growing incidence of cancer, introduction of innovative treatments, and rising drug prices.^3-5^ In the Netherlands, total spending on expensive medicines rose from €2.05 billion in 2017 to €2.5 billion in 2022 with 59% of these costs designated for cancer treatment in 2022.^6^^,^^7^

Advances in (molecular) diagnostics and an expanding range of treatment options are continuously improving cancer prognosis, resulting in higher survival rates, longer treatment durations, and enhanced quality of life.^8^ While embracing these innovations, it is crucial to use these medicines rationally and optimally to ensure patient benefits and avoid unnecessary spending. This might be hampered as new medicines often enter the market at an early stage, creating uncertainty about their real-world effectiveness, yet are usually introduced at high prices.^9-11^ Side effects or lack of effectiveness could lead to early treatment discontinuation (ETD), which can impact the overall effectiveness for patients,^12-14^ adversely affect quality of life,^15-17^ contribute to wastage of medicines, and inefficient use of the healthcare budget.^18^

Given the financial constraints in healthcare and the burden of anticancer medicines for patients, it is crucial to have insight into the number of patients affected by ETD. To date, no studies have evaluated the national budget impact of ETD for anticancer medicines. Although some reports have examined overall health care spending and cancer-related costs,^19-23^ the financial consequences of ETD remain unaddressed. Due to limited research on ETD-related costs,^16^^,^^17^ there is insufficient knowledge on the extent of ETD and its budgetary impact on healthcare. Therefore, the primary objective of this study is to analyze the number of ETD cases and associated medicine costs for the 30 anticancer medicines with the highest budget impact in the Netherlands in 2022. Additionally, this study will examine trends in ETD and its related medicine costs from 2018 to 2022.

## Methods

This real-world data study analyzes the costs of high-budget anticancer medicines and the incidence and costs of ETD of these medicines in the Netherlands. We focus on medicine costs used for the treatment of solid tumors and malignant hematological diseases.

### Context

In the Netherlands, the healthcare system is based on solidarity, with all residents having basic health insurance that covers most healthcare costs.^24^ Prescription, delivery, and budget management of expensive anticancer medicines are the responsibility of Dutch hospitals. Medical indication codes per medicine are used to ensure proper linkage to the correct condition. Oncologists register the medicine and indication code in the hospital system, which is then submitted to the health insurers. All national claims data are collected by Vektis and shared with the Dutch National Health Care Institute (Zorginstituut Nederland).

### Definitions

To analyze ETD in the Netherlands, patients were categorized into 2 groups: those who discontinued treatment early within 90 days of treatment initiation and those who continued treatment. ETD was defined as discontinuation of treatment within 90 days, with no prior use of the medicine in the preceding 365 days, and no resumption of treatment within 365 days. A gap of over 365 days between claims for the same medicine was considered a new treatment episode. The definition of ETD aligns with the national criteria used by Dutch medical oncologists (Paskwil-criteria) to assess clinically meaningful treatment effects. Treatments for metastatic disease are considered effective if they extend median overall survival by at least 12 weeks.^25^ We chose a 90-day period for ETD, as discontinuation within this timeframe makes it unlikely that the median overall survival will be extended by 12 weeks.

Within the ETD group, we examined 2 subgroups. Namely, patients surviving more than 60 days after ETD, and patients who died within 61 days after ETD or within 90 days after starting treatment.

**Figure 1.**
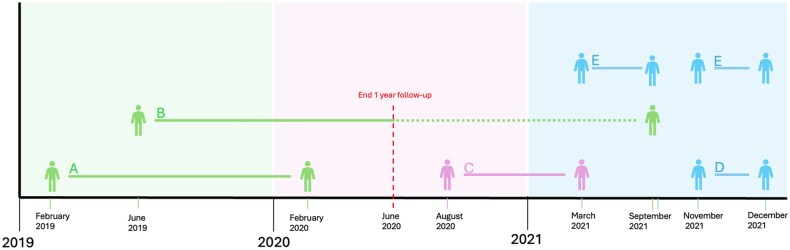
**Cost attribution based on start year of treatment**. Explanation of cost attribution per treatment based on start year of treatment. The unbroken line between 2 figures represents the treatment period, starting from the initial prescription date and ending at the last known declaration date. The colored area indicates the period to which the costs are contributed. All medication costs are attributed to the year treatment starts, even if the treatment period extends to the following year. This is illustrated by examples A, B and C. In example A treatment starts in February 2019 and continues for 1 year, all costs of this treatment are attributed to 2019. In our analysis the follow-up period is capped at one year, only costs of treatments for up to one year after the initial prescription date are tracked. This is shown in example B, because of the follow-up period of 1 year, only costs incurred up until June 2020 are considered and attributed to 2019; costs incurred after June 2020 are not included in our analysis. Example C represents a treatment started in 2020 and continued 8 months until 2021, meaning that the costs are attributed to 2020. Example D shows early treatment discontinuation (ETD) within the same year. Finally, example E shows treatment that is discontinued temporarily and restarts within 3 months after the last declaration date, this is not marked as ETD.

**Figure 2.**
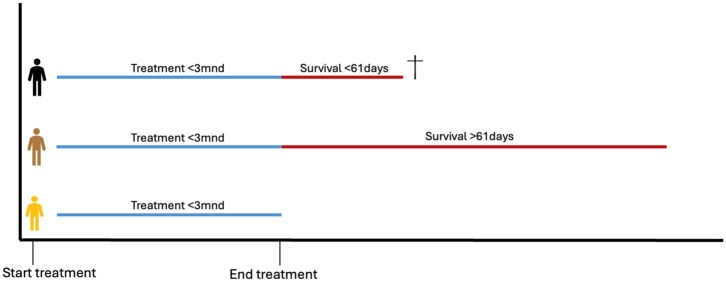
Definition subgroups. Overview of subgroups used in this study. The blue line indicates treatment shorter than 3 months. The red line indicates survival period after last declaration date. The bottom figure represents the total ETD group. The total ETD group is separated into 2 subgroups, namely survivors and deaths. ETD survivors, represented by the figure in the middle, survive longer than 61 days after the last declaration date. ETD deaths, represented by the top figure, die within 61 days after the last declaration date.

Overall annual medicine costs and treatment numbers reflect all treatment initiations, including both completed treatments and treatments discontinued early, within a 1-year follow-up period. To assess the financial burden, costs were reallocated to the year of treatment initiation, even if incurred in the subsequent year (see Figures 1 and 2). This approach enables a comprehensive assessment of the budgetary impact of treatment initiation and early discontinuation, allowing for comparisons between treatments with ETD and treatments that were continued.

### Medicine selection

In this study, we selected 30 anticancer medicines with the highest budget impact in 2022, the most recent year with a nearly complete dataset in the following year. Three anticancer medicines were excluded from entering the top 30 due to treatment regimens shorter than 90 days. The excluded medicines were axicabtagen ciloleucel (single dose), ipilimumab (4 cycles of 3 weeks) and pemetrexed (3 cycles of 3 weeks). Additionally, we analyzed claims data for all included medicines from 2018 to 2022, acknowledging that not all were reimbursed before and in 2018. For medicines approved for both malignant and non-malignant diseases, only malignant indications along with their associated costs and treatments were included, based on the specific indication codes.

### Dataset

This study utilized healthcare performance declaration data (Zorg Prestatie Declaratie-data) which encompasses all insurance claims for high-cost medicines in the Netherlands. These data were supplied by the Dutch National Health Care Institute in November 2024. The anonymous dataset includes claims data for 30 selected anticancer medicines from patients who started treatment between January 1, 2018 and December 31, 2022. Extracted variables included indication codes, yearly declared costs per medicine and indication, number of patients, and identification of patients who died within 61 days after their last declaration date or within 90 days after treatment start. For hospital-administered medicines, the declaration date matches the last treatment date, while for oral medicines, patients may start treatment on a date later than the declaration date. Based on the first and last declaration dates, the treatment duration was determined. Data on subsequent treatments were unavailable.

### Analysis

To calculate the overall costs per medicine (*C*_medicine_), we summed all costs for oncological and hematological indications codes (*C*_indication_) across all patients (Formula S1). Costs incurred in a subsequent year but within 365 days after treatment initiation were attributed to the treatment start year (Figure S1). Annual expenditure (*C*_total_) is determined by summing the overall costs per medicine per year (Formula S2).

The same formulas are applied to calculate total ETD costs and costs in the 2 subgroups: patients who survived more than 60 days after ETD survivor and patients who died within 61 days after ETD. This method was also used to determine the number of treatments per indication, per medicine, overall, and for both subgroups.

### Ethical approval

This study does not fall within the scope of the “WMO” (Dutch Medical Research Involving Human Subject Act); therefore, ethical review of the study was not needed. Data were stored and shared according to the European general data protection regulation.

## Results

### Selection of medicines

Thirty medicines were selected for analysis. Indications classified as malignant, but addressing non-cancer-related issues, such as treatment-induced complications (eg, radionecrosis), were excluded on an individual basis. Three medicines—durvalumab, encorafenib, and binimetinib—were not reimbursed in 2018, meaning no data were available for these treatments that year. The characteristics of the selected medicines are provided in Table SA.

### Total early treatment discontinuation and costs in 2022

In 2022, the total annual expenditure (overall costs) on the 30 selected medicines amounted to €783 million. The total ETD costs for these medicines were €77 million, representing 9.9% of the overall costs. Immunotherapies dominated both overall and ETD costs. The highest ETD costs were observed for pembrolizumab (€26 million), nivolumab (€7.9 million) and daratumumab (€7.3 million) together accounting for 53.3% of the total ETD costs (Table 1). For all early-discontinued medicines except osimertinib, more ETD costs were incurred by patients who survived beyond 60 days than by those who died within that period.

**Table 1.** Overview of costs and number of treatments of top 30 highest-budget impact anticancer medicines in the Netherlands in 2022.

Medicines	Overall costs of all treatments^a^ (x €1000)	Overall no. treatments^a^	Costs ETD total (x €1000)	No. ETD treatments	% of total costs	Costs ETD survivors (x €1000)	No. ETD survivors	% of total costs	Costs ETD deaths (x €1000)	No. ETD deaths	% of total costs
**PEMBROLIZUMAB**	€ 198,091	4460	€ 26,011	1812	13.1%	€ 16,749	1035	8.5%	€ 9,262	777	4.7%
**DARATUMUMAB**	€ 121,503	1545	€ 7,335	256	6.0%	€ 5,334	163	4.4%	€ 2,001	93	1.6%
**NIVOLUMAB**	€ 73,893	2464	€ 7,906	1007	10.7%	€ 6,018	730	8.1%	€ 1,887	277	2.6%
**DURVALUMAB**	€ 50,487	805	€ 3,871	228	7.7%	€ 3,534	201	7.0%	€ 336	27	0.7%
**OSIMERTINIB**	€ 32,476	499	€ 1,229	82	3.8%	€ 702	37	2.2%	€ 527	45	1.6%
**ENZALUTAMIDE**	€ 29,729	1267	€ 2,114	293	7.1%	€ 1,598	212	5.4%	€ 515	81	1.7%
**PERTUZUMAB**	€ 21,572	1237	€ 1,726	224	8.0%	€ 1,607	207	7.5%	€ 119	17	0.6%
**IBRUTINIB**	€ 20,707	411	€ 1,551	103	7.5%	€ 932	60	4.5%	€ 619	43	3.0%
**TRASTUZUMAB EMTANSINE**	€ 18,362	521	€ 1,534	138	8.4%	€ 1,390	120	7.6%	€ 144	18	0.8%
**ABIRATERON**	€ 17,639	1764	€ 2,106	427	11.9%	€ 1,637	316	9.3%	€ 468	111	2.7%
**PALBOCICLIB**	€ 17,340	1003	€ 1,204	247	6.9%	€ 1,008,	196	5.8%	€ 196	51	1.1%
**POMALIDOMIDE**	€ 15,134	279	€ 1,642	103	10.9%	€ 1,109	61	7.3%	€ 533	42	3.5%
**TRAMETINIB**	€ 14,331	388	€ 1,685	144	11.8%	€ 1,178	102	8.2%	€ 507	42	3.5%
**DABRAFENIB**	€ 14,019	394	€ 1,763	148	12.6%	€ 1,243	106	8.9%	€ 520	42	3.7%
**TRASTUZUMAB**	€ 12,511	2135	€ 207	156	1.7%	€ 142	96	1.1%	€ 65	60	0.5%
**ENCORAFENIB**	€ 12,432	385	€ 1,652	143	13.3%	€ 956	79	7.7%	€ 696	64	5.6%
**BEVACIZUMAB**	€ 11,803	2332	€ 1,660	889	14.1%	€ 1,345	696	11.4%	€ 315	193	2.7%
**RITUXIMAB**	€ 10,966	3784	€ 1,734	1135	15.8%	€ 1,524	927	13.9%	€ 209	208	1.9%
**RUXOLITINIB**	€ 10,930	323	€ 393	52	3.6%	€ 285	37	2.6%	€ 108	15	1.0%
**VENETOCLAX**	€ 10,429	383	€ 758	153	7.3%	€ 465	95	4.5%	€ 293	58	2.8%
**CARFILZOMIB**	€ 10,280	227	€ 1,278	90	12.4%	€ 858	52	8.4%	€ 419	38	4.1%
**OLAPARIB**	€ 10,162	241	€ 673	61	6.6%	€ 623	55	6.1%	€ 49	6	0.5%
**NIRAPARIB**	€ 9,328	251	€ 1,181	84	12.7%	€ 1,165	83	12.5%	€ 16	1	0.2%
**CABAZITAXEL**	€ 8,768	601	€ 2,177	287	24.8%	€ 1,590	192	18.1%	€ 586	95	6.7%
**BINIMETINIB**	€ 8,615	265	€ 1,001	88	11.6%	€ 580	49	6.7%	€ 420	39	4.9%
**RADIUM RA-223 DICHLORIDE**	€ 7,643	366	€ 1,762	150	23.1%	€ 1,331	109	17.4%	€ 430	41	5.6%
**ALECTINIB**	€ 4,690	93	€ 245	19	5.2%	€ 150	12	3.2%	€ 94	7	2.0%
**LENALIDOMIDE**	€ 4,562	1674	€ 735	453	16.1%	€ 548	311	12.0%	€ 186	142	4.1%
**DASATINIB**	€ 2,615	179	€ 206	39	7.9%	€ 173	31	6.6%	€ 33	8	1.3%
**NILOTINIB**	€ 1,695	67	€ 98	14	5.8%	€ 98	14	5.8%	N.A.	0	0.0%
**Total**	€ 782,729	30343	€ 77,452	9025	9.9%	€ 55,885	6384	7.1%	€ 21,567	2641	2.8%

a Overall costs and overall no. include all treatments and costs per medicine. Costs have been rounded to the nearest thousand euros.

Abbreviations: No., number; ETD, early treatment discontinuation.

In 2022, 30 343 treatments were administered with the selected high-cost medicines, of which 9025 treatments (29.7%) were discontinued early. In that year, the medicines with the highest number of ETD were pembrolizumab (*n* = 1812), rituximab (*n* = 1135), and nivolumab (*n* = 1007), collectively accounting for 43.8% of all ETDs (Table 1).

A high degree of heterogeneity was observed between medicines. Treatments commonly used in later lines of therapy, such as cabazitaxel and radium-223 dichloride, showed the highest proportions of ETD (over 40%), whereas trastuzumab, generally used in earlier treatment lines, showed the lowest proportion of ETD (less than 10%). A detailed overview of costs and ETD per medicine over the years is provided in Table SB.

### Total costs of early treatment discontinuation between 2018 and 2022

Between 2018 and 2022, overall costs for the selected medicines rose from €616 million in 2018 to €783 million in 2022—an increase of 27.1%—with a peak of €832 million in 2021. Between 2018 and 2019, the number of treatments rose by 3336, driven largely by increased use of already reimbursed medicines like pembrolizumab and daratumumab. Only 22.2% of the €150 million cost increase resulted from the addition of durvalumab, encorafenib, and binimetinib to the reimbursed package. Conversely, for other medicines, particularly lenalidomide and abiraterone, the declared price decreased over time. This price reduction contributed to the decline in total expenditure in 2022 compared to 2021. A full overview is provided in Table SB.

**Figure 3.**
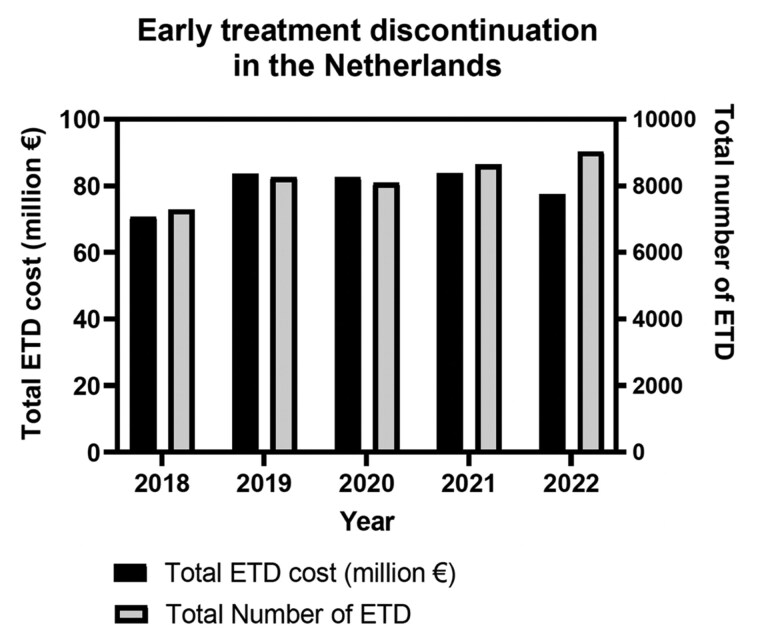
Overview of the costs and number of ETD in the years 2018 until 2022.

During this period, total ETD costs increased from €71 million in 2018 to €77 million in 2022, a 9.6% increase. Meanwhile, the annual ETD share of the total costs declined from 11.5% in 2018 to 9.9% in 2022. ETD costs for patients surviving more than 60 days after ETD increased from €51 million in 2018 to €56 million in 2022, representing 8.3% and 7.1% of total expenditures, respectively. ETD costs for patients who died within 61 days after ETD rose from €20 million in 2018 to €22 million in 2022, accounting for 3.2% of total expenditure in 2018 and 2.8% in 2022 (Table 2; Figure 3).

**Table 2.** Overview of costs and changes over time from 2018 to 2022.

Years	Overall costs of all treatments (x €1,000)	Total ETD cost (x €1,000)	**% Δ** ^a^	% cost ETD of total costs	Costs ETD survivors (x €1,000)	**% Δ** ^a^	% cost ETD survivors of total costs	Costs ETD deaths (x €1,000)	**% Δ** ^a^	% cost ETD deaths of total costs
**2022**	€ 782,729	€ 77,452	−7.5%	9.9%	€ 55,885	−7.0%	7.1%	€ 21,567	−8.9%	2.8%
**2021**	€ 832,235	€ 83,757	1.5%	10.1%	€ 60,063	1.3%	7.2%	€ 23,694	1.8%	2.9%
**2020**	€ 796,092	€ 82,549	−1.2%	10.4%	€ 59,274	−1.6%	7.5%	€ 23,274	−0.1%	2.9%
**2019**	€ 765,702	€ 83,553	18.3%	10.9%	€ 60,265	18.5%	7.9%	€ 23,287	17.6%	3.0%
**2018**	€ 615,925	€ 70,649	N.A.^b^	11.5%	€ 50,854	N.A.^b^	8.3%	€ 19,795	N.A.^b^	3.2%

Costs in this table are the costs of all 30 medicines of Table 1 summed to a yearly total per group per year. Costs have been rounded to the nearest thousand euros.

a % Δ Represents the change in ETD costs in comparison with the previous year. This change is presented in percentages.

b Data not available. data before 2018 are not collected.

Abbreviations: ETD, early treatment discontinuation; N.A., not available.

### Total early treatment discontinuations between 2018 and 2022

Over time, the number of treatments initiated with selected high-budget impact medicines increased by 34.0% from 22 639 in 2018 to 30 343 in 2022 (Table 3). Between 2018 and 2022, the total number of ETDs increased from 7287 in 2018 to 9025 in 2022. Despite this rise, the proportion of ETDs relative to the overall number of treatments decreased from 32.2% in 2018 to 29.7% in 2022. The results highlight a notable overall increase in the number of treatments with high-budget anticancer medicines, with a slight decrease in the proportion of ETD. Additionally, the percentage of ETD followed by death, relative to the total number of treatments, declined from 9.7% to 8.7% across the study period (Table 3; Figure 3).

**Table 3.** Overview of number of treatments and changes over time from 2018 to 2022.

Years	Overall No. of all treatments	Total No. ETD treatments	**% Δ** ^a^	% No. ETD treatments of total	No. ETD survivors	% delta	% No. ETD survivors of total	No. ETD deaths	% delta	% No. of ETD deaths of total
**2022**	30343	9025	4.5%	29.7%	6384	5.1%	21.0%	2641	3.1%	8.7%
**2021**	28835	8635	6.8%	30.0%	6073	6.6%	21.1%	2562	7.3%	8.9%
**2020**	26308	8084	−2.2%	30.7%	5696	-2.8%	21.7%	2388	−0.6%	9.1%
**2019**	25975	8262	13.4%	31.8%	5860	15.4%	22.6%	2402	8.7%	9.3%
**2018**	22639	7287	N.A.^b^	32.2%	5078	N.A.^b^	22.4%	2209	N.A.^b^	9.8%

Number of treatments of all 30 medicines summed to a yearly total per group. Costs have been rounded to the nearest thousand euros.

a % Δ Represents the change in ETD number of treatments in comparison with the previous year. This change is presented in percentages.

b Data not available, data before 2018 are not collected.

Abbreviations: No., number; ETD, early treatment discontinuation; N.A., not available.

## Discussion

This study provides a comprehensive analysis of ETD and its associated costs for 30 high-budget anticancer medicines in the Netherlands. To our knowledge, it is the first national-level study quantifying both the number of ETD cases and their associated costs for anticancer medicines with the highest budget impact. The findings signal that a significant proportion of healthcare budget is spend on the use of these high-cost medicines among patients who need to discontinue treatment early. In our baseline year, 2022, nearly one in 3 anticancer medicines were discontinued within 90 days, representing approximately €77 million, about 10% of total yearly expenditures on these medicines. Between 2018 and 2022, both the number of ETDs and their associated costs increased, while their share of both numbers and costs declined slightly, underscoring a persistent clinical and financial relevance of ETD.

While our findings suggest room for improvement in the use of high-cost anticancer medicines, the primary aim of this study is to quantify ETD and examine trends over time. Given the study design, we were unable, and not intending, to assess clinical appropriateness at the individual patient level (eg, through review of medical records). Instead, our focus was to identify patterns and cost implications of ETD at the population level, providing insight into where improved patient selection and treatment optimization may have the greatest impact. Importantly, ETD should not be interpreted as a single negative outcome or a uniform indicator of inefficiency, rather it reflects the outcome of an interplay between complex heterogeneous clinical circumstances. Some ETDs occur because treatments are not effective for all patients, may serve as last resort option in patients with good performance status, or lose effectiveness near the end of life, as an unavoidable consequence of disease progression. Other cases reflect end-of-life overtreatment in patients with poor performance status or after multiple, rapidly failing prior therapies, where benefit is unlikely. ETD may be appropriate when lack of benefit is recognized in time, sparing patients with unnecessary toxicity, allowing them to receive subsequent treatment or best supportive care. Finally, ETD may occur when side effects become too severe for patients to continue treatment, which may improve over time or, in some cases, contribute to early death. Altogether, these heterogenous reasons voor ETD carry different implications for patient outcomes, healthcare efficiency, and the policy decisions needed to address them. Not all ETD is preventable within the current state of medical science. Our distinction between patients who survived more than 60 days after discontinuation and those who died within 61 days was the only categorization our data could substantiate and serves as an initial step toward differentiating between more and less appropriate ETD.

The data show that in 2022 pembrolizumab, daratumumab, and nivolumab together represented a disproportionate share (more than 50%) of total- and ETD-related costs. Most patients who discontinued these treatments early, survived more than 60 days after ETD, suggesting that these ETDs do not necessarily reflect inappropriate care, or are used in earlier lines of therapy. However, also a substantial proportion of these patients (27.5-42.8%) died shortly after ETD, highlighting a need for improved patient selection to better support patients’ outcomes and reduce avoidable costs. In terms of frequency, pembrolizumab, bevacizumab, and rituximab had the highest absolute ETD numbers, probably reflecting toxicity- and/or progression-driven discontinuation in earlier lines of therapy. Cabazitaxel and radium-223 dichloride showed the highest percentages of ETD, probably reflecting disease progression near the end of life or last resort overtreatment, given the line of treatment in which they are used. These data underscore substantial clinical heterogeneity of ETD.

Reducing avoidable ETD requires better alignment between treatment initiation, patient characteristics, and expected benefit. Current research on predictive factors, such as tumor response biomarkers and treatment-related toxicities,^26-30^ remains limited in its ability to predict ETD at the individual level.^31^^,^^32^ Complementary strategies, such as dose optimization, interval extension, and supportive care interventions, can further mitigate toxicity-driven ETD and improve treatment adherence.^33^^,^^34^ Finally, excluding patients unlikely to benefit, based on predictive factors, may support more appropriate and effective end-of-life care.^35-37^

A major strength of this study lies in its use of real-world, comprehensive national claims data, covering approximately 98% of all Dutch claims data. The data extraction from November 2024 revealed stable treatment volumes in 2022 and only a minimal increase (+0.01%) compared to the august 2024 version. Costs from 2023 for treatments initiated in 2022 increased by just 0.6%, supporting completeness of the data.

Nevertheless, several limitations should be acknowledged. Restricting follow-up to one year likely led to underestimation of long-term treatment costs and overestimation of ETD’s relative share, as around 20% of total costs for long-term treatments may have been excluded. The absence of linked clinical data, including outcomes, performance status, comorbidities, and biomarkers, limits our ability to disentangle the relative contributions of drug-related, disease-related, and non-tumor-related factors. For example, patients treated with immune checkpoint inhibitors like pembrolizumab may achieve durable benefit despite early discontinuation; such cases cannot be identified in claims data alone.^38^

Moreover, part of the study period coincides with the COVID-19 pandemic (2020-2021), during which treatment initiation and continuation were influenced by infection risk, hospital capacity, and more stringent patient selection criteria, potentially affecting ETD patterns.

Cost estimates are based on declared medicine expenditures and exclude confidential rebates and risk-sharing agreements. As these arrangements are not publicly available, reported figures should be interpreted as upper-bound estimates. Finally, we did not adjust for inflation or relate costs to gross domestic product over the same period,^39^ which may affect the estimation of budgetary impact.

This study did not aim to provide a complete overview of ETD-related healthcare costs, but to generate an initial estimate of ETD-related medicine expenditures for high-cost anticancer medicines only. To fully characterize the true costs associated with ETD of high-cost anticancer therapies, future analyses would need to account for additional cost components, including concurrent (chemo)therapy, supportive care (eg, antiemetics, growth factors, transfusions), broader healthcare utilization (eg, hospitalizations, emergency visits, imaging, outpatient consultations), and relevant clinical outcomes. Importantly, further research is needed into the root causes of ETD before robust policy recommendations can be formulated, and it may be highly valuable for oncologists to share benchmarking data (“mirror information”) on treatment durations and discontinuation reasons across centers, to learn from real-world practice and inform better access policy.

## Conclusion

In conclusion, approximately 30% of anticancer treatments in the Netherlands are discontinued within 90 days, representing about 10% of total medicine expenditure in the first treatment year. While these findings signal a substantial burden, they should not be interpreted as evidence of inefficiency in all cases. ETD encompasses a heterogeneous mix of appropriate, inevitable, and avoidable discontinuations. Distinguishing between these categories is essential to guide more effective clinical, analytical, and policy responses. The persistent burden of ETD highlights the urgent need for research that improves patient selection and treatment optimization to enhance efficiency and rational use of high-cost anticancer therapies.

## Supplementary Material

oyaf436_Supplementary_Data

## Data Availability

Aggregated data generated and analyzed during this study are available from the corresponding author upon reasonable request. The underlying data were obtained from Vektis, the Dutch national healthcare claims database, and cannot be shared by the authors due to legal and contractual restrictions. Access to Vektis data may be granted to qualified researchers upon request and subject to approval by Vektis and applicable data protection regulations.
