# Supplementary material for: Cost implications of early treatment discontinuation in cancer: a real-world data analysis
Source: Oncologist. 2026 Feb 25;31(4):oyaf436. doi: 10.1093/oncolo/oyaf436 (PMC12995430; doi:10.1093/oncolo/oyaf436)
Supplement: oyaf436_Supplementary_Data [file oyaf436_supplementary_data.zip › 2025111 Supplementary Tables_Vroegstoppers_.docx]

**Supplementary Tables**

| **Medicines** | **Drug class** | **Working mechanism** | **Biomarker availability** | **EU Marketing authorization** | **First Dutch reimbursement date** | **Treatment schedule** |
| --- | --- | --- | --- | --- | --- | --- |
| Abirateron | Androgen biosynthesis inhibitor | Targeted | None | 05/09/2011 | 01/02/2017 | Once daily |
| Alectinib | Tyrosin kinase inhibitor | Targeted ALK en RET | Available | 16/02/2017 | 01/05/2017 | Twice daily |
| Bevacizumab | Monoclonal antibody | Targeted VEGF remmer | None | 12/01/2005 | 01/01/2017 | d1 q14 or q21 sc |
| Binimetinib | Tyrosin kinase inhibitor | Targeted MEK | Available | 20/09/2018 | 01/12/2018 | Twice daily |
| Cabazitaxel | Taxanes | Chemotherapy | None | 17/03/2011 | 01/01/2017 | d1q21 iv |
| Carfilzomib | Proteasome inhibitor | Targeted 20s-eiwit subunit | None | 19/11/2015 | 01/01/2017 | d1,2,8,9,15 en 16 q28 iv |
| Dabrafenib | Tyrosin kinase inhibitor | Targeted BRAF600 | Available | 26/08/2013 | 01/01/2017 | Twice daily |
| Daratumumab | Monoclonal antibody | Targeted CD38 | None | 20/05/2016 | 01/04/2017 | Weekly - monthly |
| Dasatinib | Tyrosin kinase inhibitor | Targeted BCR-ABL | Available | 20/11/2006 | 01/01/2017 | Once daily |
| Durvalumab | Immune checkpoint inhibitor | Immune checkpoint inhibitor PDL1 | None | 21/09/2018 | 01/09/2019 | 2 weekly - monthly |
| Encorafenib | Tyrosin kinase inhibitor | Targeted BRAF600 | Available | 19/09/2018 | 01/12/2018 | Once daily |
| Enzalutamide | Androgen receptor signalling inhibitor | Targeted | None | 21/06/2013 | 01/01/2017 | Once daily |
| Ibrutinib | Tyrosin kinase inhibitor | Targeted | None | 21/10/2014 | 01/01/2017 | Once daily |
| Lenalidomide | Immunosuppressants | Other | None | 14/06/2007 | 01/01/2017 | Once daily |
| Nilotinib | Tyrosin kinase inhibitor | Targeted BCR-ABL | Available | 19/11/2007 | 01/01/2017 | Twice daily |
| Niraparib | PARP inhibitor | Targeted BRCA | Available | 16/11/2017 | 01/06/2018 | Once daily |
| Nivolumab | Immune checkpoint inhibitor | Immune checkpoint inhibitor PD1 | Available | 19/06/2015 | 01/01/2017 | 2 weekly - monthly |
| Olaparib | PARP inhibitor | Targeted BRCA | Available | 16/12/2014 | 01/01/2017 | Twice daily |
| Osimertinib | Protein kinase inhibitor | Targeted EGFR | Available | 01/02/2016 | 01/01/2017 | Once daily |
| Palbociclib | CDK4/6 inhibitor | Cell cycle | None | 09/11/2016 | 01/06/2017 | orally,d1-21, q28  3 |
| Pembrolizumab | Immune checkpoint inhibitor | Immune checkpoint inhibitor PD1 | Available | 17/07/2015 | 01/07/2017 | metastatic up to progression, every 3 to 6 weeks, expect NSCLC neo-adjuvant |
| Pertuzumab | Monoclonal antibody | Targeted HER2 | Available | 04/03/2013 | 01/01/2017 | 3 weekly |
| Pomalidomide | Immunosuppressants | other | None | 05/08/2013 | 01/01/2017 | Different schemes |
| Radium RA-223 dichloride | Radiopharmaceutical | Alfa-emitter | None | 13/11/2013 | 01/01/2017 | 4 weekly |
| Rituximab | Monoclonal antibody | Targeted CD20 | Available | 02/06/1998 | 01/01/2017 | 2 weekly – 3 monthly |
| Ruxolitinib | Protein kinase inhibitor | Other JAK1 en 2 | None | 23/08/2012 | 01/01/2017 | Twice daily |
| Trametinib | Tyrosin kinase inhibitor | Targeted MEK | Available | 30/06/2014 | 01/01/2017 | Once daily |
| Trastuzumab | Monoclonal antibody | Targeted HER2 | Available | 28/08/2000 | 01/01/2017 | 3 weekly |
| Trastuzumab emtansine | Antibody drug conjugate | Targeted HER2 | Available | 15/11/2013 | 01/01/2017 | 3 weekly |
| Venetoclax | BCL-2-inhibitor | Targeted BCL-2 | Avialable | 4/12/2016 | 01/02/2017 | Once daily |

**Supplementary table A:** Overview of baseline characteristics of included medicines. D = day a medicine is taken per cycle, q = duration of a cycle in days

| **2018** | | | | | | | | | |
| --- | --- | --- | --- | --- | --- | --- | --- | --- | --- |
| **Anti-cancer medicines** |  | **Overall costs**  **(x 1,000)** | **Overall No.**  **Treatments** | **Costs ETD total**  **(x 1,000)** | **No.**  **Treatments ETD total** | **Costs EDT survivors**  **(x 1,000)** | **No. EDT survivors** | **Costs ETD deaths**  **(x 1,000)** | **No. ETD deaths** |
| PEMBROLIZUMAB |  | € 100,371 | 1961 | € 12,242 | 813 | € 7,187 | 409 | € 5,054 | 404 |
| LENALIDOMIDE |  | € 70,966 | 1499 | € 4,044 | 350 | € 2,862 | 235 | € 1,181 | 115 |
| TRASTUZUMAB |  | € 46,235 | 2233 | € 759 | 168 | € 500 | 99 | € 258 | 69 |
| NIVOLUMAB |  | € 45,305 | 1854 | € 7,844 | 1011 | € 5,271 | 604 | € 2,573 | 407 |
| ENZALUTAMIDE |  | € 40,978 | 1590 | € 3,366 | 411 | € 2,492 | 290 | € 873 | 121 |
| BEVACIZUMAB |  | € 40,967 | 2463 | € 5,432 | 910 | € 4,454 | 721 | € 978 | 189 |
| PALBOCICLIB |  | € 32,214 | 1385 | € 2,837 | 368 | € 2,398 | 303 | € 438 | 65 |
| RITUXIMAB |  | € 28,818 | 3670 | € 4,265 | 1063 | € 3,789 | 866 | € 476 | 197 |
| DARATUMUMAB |  | € 27,099 | 323 | € 4,344 | 123 | € 2,788 | 63 | € 1,556 | 60 |
| PERTUZUMAB |  | € 26,285, | 1152 | € 5,338 | 430 | € 5,215 | 415 | € 123 | 15 |
| ABIRATERON |  | € 18,684 | 878 | € 3,106 | 381 | € 2,339 | 266 | € 767 | 115 |
| IBRUTINIB |  | € 16,259 | 303 | € 1,385 | 90 | € 861 | 53 | € 523 | 37 |
| DABRAFENIB |  | € 15,807 | 438 | € 2,406 | 163 | € 1,449 | 96 | € 957 | 67 |
| TRAMETINIB |  | € 15,622 | 434 | € 2,568 | 166 | € 1,494 | 97 | € 1,073 | 69 |
| CARFILZOMIB |  | € 13,187 | 297 | € 1,370 | 103 | € 813 | 48 | € 557 | 55 |
| OSIMERTINIB |  | € 12,468 | 204 | € 884 | 53 | € 415 | 23 | € 468 | 30 |
| RUXOLITINIB |  | € 11,226 | 292 | € 456 | 53 | € 361 | 39 | € 94 | 14 |
| RADIUM RA-223 DICHLORIDE |  | € 10,250 | 467 | € 2,091 | 167 | € 1,732 | 131 | € 359 | 36 |
| CABAZITAXEL |  | € 9,292 | 411 | € 2,412 | 205 | € 1,794 | 136 | € 618 | 69 |
| POMALIDOMIDE |  | € 7,674 | 145 | € 1,263 | 70 | € 849 | 42 | € 414 | 28 |
| ALECTINIB - (L01ED03) |  | € 6,896 | 113 | € 304 | 21 | € 199 | 11 | € 104 | 10 |
| TRASTUZUMAB EMTANSINE |  | € 5,493 | 137 | € 809 | 57 | € 538 | 36 | € 270 | 21 |
| DASATINIB |  | € 4,258 | 129 | € 261 | 35 | € 235 | 30 | € 26 | 5 |
| OLAPARIB |  | € 3,815 | 88 | € 369 | 25 | € 358 | 24 | € 11 | 1 |
| NILOTINIB |  | € 3,644 | 117 | € 187 | 27 | € 183 | 25 | € 4 | 2 |
| NIRAPARIB |  | € 1,467 | 31 | € 232 | 12 | € 232 | 12 | € 0 | 0 |
| VENETOCLAX |  | € 632 | 25 | € 63 | 12 | € 35 | 4 | € 27 | 8 |
| DURVALUMAB |  | N.A. | N.A. | N.A. | N.A. | N.A. | N.A. | N.A. | N.A. |
| ENCORAFENIB |  | N.A. | N.A. | N.A. | N.A. | N.A. | N.A. | N.A. | N.A. |
| BINIMETINIB |  | N.A. | N.A. | N.A. | N.A. | N.A. | N.A. | N.A. | N.A. |
| **Totaal** |  | **€ 615,925** | **22639** | **€ 70,649** | **7287** | **€ 50,854** | **5078** | **€ 19,795** | **2209** |
|  | | | | | | | | | |
|  | | | | | | | | | |
| **2019** | | | | | | | | | |
| **Anti-cancer medicines** |  | **Overall costs**  **(x 1,000)** | **Overall No. Treatments** | **Costs ETD total**  **(x 1,000)** | **No.**  **Treatments ETD total** | **Costs EDT survivors**  **(x 1,000)** | **No. EDT survivors** | **Costs ETD deaths**  **(x 1,000)** | **No. ETD deaths** |
|  |  |  |  |  |  |  |  |  |  |
| PEMBROLIZUMAB |  | € 149,113 | 3160 | € 17,681 | 1247 | € 10,494 | 643 | € 7,187 | 604 |
| LENALIDOMIDE |  | € 77,067 | 1637 | € 4,667 | 403 | € 3,452 | 282 | € 1,214 | 121 |
| NIVOLUMAB |  | € 75,276 | 2488 | € 8,821 | 1056 | € 6,413 | 698 | € 2,407 | 358 |
| DARATUMUMAB |  | € 74,800 | 828 | € 7,274 | 211 | € 4,156 | 108 | € 3,117 | 103 |
| ENZALUTAMIDE |  | € 37,135 | 1483 | € 3,291 | 398 | € 2,381 | 272 | € 910 | 126 |
| BEVACIZUMAB |  | € 34,701 | 2248 | € 5,308 | 886 | € 4,383 | 707 | € 924 | 179 |
| TRASTUZUMAB |  | € 30,258 | 2111 | € 534 | 176 | € 423 | 130 | € 110 | 46 |
| PERTUZUMAB |  | € 27,987 | 1289 | € 4,850 | 418 | € 4,738 | 404 | € 112 | 14 |
| ABIRATERON |  | € 27,598 | 1156 | € 3,295 | 410 | € 2,460 | 284 | € 834 | 126 |
| PALBOCICLIB |  | € 25,247 | 1232 | € 2,124 | 327 | € 1,869 | 281 | € 254 | 46 |
| RITUXIMAB |  | € 23,896 | 3736 | € 3,590 | 1107 | € 3,155 | 896 | € 434 | 211 |
| DURVALUMAB |  | € 23,563 | 464 | € 2,375 | 142 | € 2,344 | 137 | € 30 | 5 |
| IBRUTINIB |  | € 22,505 | 405 | € 1,454 | 99 | € 836 | 54 | € 618 | 45 |
| OSIMERTINIB |  | € 15,208 | 235 | € 829 | 51 | € 197 | 15 | € 632 | 36 |
| DABRAFENIB |  | € 15,028 | 428 | € 2,462 | 172 | € 1,728 | 117 | € 733 | 55 |
| TRAMETINIB |  | € 13,999 | 423 | € 2,317 | 173 | € 1,679 | 120 | € 638 | 53 |
| RUXOLITINIB |  | € 11,484 | 302 | € 296 | 42 | € 239 | 33 | € 56 | 9 |
| CABAZITAXEL |  | € 11,194 | 485 | € 3,122 | 244 | € 2,251 | 165 | € 870 | 79 |
| CARFILZOMIB |  | € 9,412 | 215 | € 1,280 | 87 | € 909 | 52 | € 371 | 35 |
| POMALIDOMIDE |  | € 9,368 | 170 | € 1,568 | 79 | € 1,057 | 49 | € 510 | 30 |
| TRASTUZUMAB EMTANSINE |  | € 8,299 | 181 | € 817 | 60 | € 570 | 38 | € 246 | 22 |
| RADIUM RA-223 DICHLORIDE |  | € 8,109 | 400 | € 2,299 | 184 | € 1,772 | 134 | € 526 | 50 |
| ALECTINIB |  | € 6,034 | 99 | € 171 | 16 | € 118 | 10 | € 53 | 6 |
| OLAPARIB |  | € 5,475 | 139 | € 656 | 45 | € 611 | 41 | € 45 | 4 |
| ENCORAFENIB |  | € 5,112 | 138 | € 652 | 60 | € 451 | 46 | € 200 | 14 |
| BINIMETINIB |  | € 4,741 | 137 | € 580 | 58 | € 414 | 45 | € 166 | 13 |
| DASATINIB |  | € 4,212 | 139 | € 205 | 29 | € 159 | 23 | € 46 | 6 |
| NIRAPARIB |  | € 3,000 | 94 | € 727 | 47 | € 727 | 47 | € 0 | 0 |
| NILOTINIB |  | € 2,971 | 99 | € 127 | 23 | € 122 | 21 | € 5 | 2 |
| VENETOCLAX |  | € 2,896 | 54 | € 170 | 12 | € 145 | 8 | € 24 | 4 |
| **Totaal** |  | **€ 765,702** | **25975** | **€ 83,553** | **8262** | **€ 60,265** | **5860** | **€ 23,287** | **2402** |
|  | | | | | | | | | |
|  | | | | | | | | | |
|  | | | | | | | | | |
| **2020** | | | | | | | | | |
| **Anti-cancer medicines** |  | **Overall costs**  **(x 1,000)** | **Overall No. Treatments** | **Costs ETD total**  **(x 1,000)** | **No.**  **Treatments ETD total** | **Costs EDT survivors**  **(x 1,000)** | **No. EDT survivors** | **Costs ETD deaths**  **(x 1,000)** | **No. ETD deaths** |
| PEMBROLIZUMAB |  | € 175,969 | 3757 | € 22,788 | 1571 | € 14,489 | 879 | € 8,298 | 692 |
| DARATUMUMAB |  | € 83,266 | 962 | € 7,906 | 224 | € 5,379 | 130 | € 2,526 | 94 |
| LENALIDOMIDE |  | € 60,946 | 1410 | € 4,519 | 397 | € 3,139 | 263 | € 1,380 | 134 |
| NIVOLUMAB |  | € 59,228 | 1925 | € 6,250 | 808 | € 4,769 | 575 | € 1,480 | 233 |
| DURVALUMAB |  | € 39,955 | 644 | € 2,514 | 168 | € 2,299 | 154 | € 214 | 14 |
| OSIMERTINIB |  | € 38,410 | 566 | € 1,577 | 100 | € 822 | 46 | € 754 | 54 |
| ENZALUTAMIDE |  | € 35,056 | 1432 | € 2,725 | 353 | € 1,885 | 229 | € 840 | 124 |
| BEVACIZUMAB |  | € 29,337 | 2279 | € 4,142 | 808 | € 3,378 | 634 | € 763 | 174 |
| ABIRATERON |  | € 28,501 | 1173 | € 2,800 | 339 | € 2,126 | 244 | € 674 | 95 |
| PERTUZUMAB |  | € 28,036 | 1213 | € 3,350 | 297 | € 3,269 | 286 | € 80 | 11 |
| IBRUTINIB |  | € 23,318 | 425 | € 1,293 | 92 | € 780 | 53 | € 513 | 39 |
| PALBOCICLIB |  | € 21,143 | 1139 | € 1,779 | 308 | € 1,521 | 257 | € 257 | 51 |
| RITUXIMAB |  | € 18,738 | 3724 | € 2,745 | 1065 | € 2,347 | 841 | € 398 | 224 |
| TRASTUZUMAB |  | € 18,569 | 1821 | € 380 | 180 | € 271 | 123 | € 109 | 57 |
| CABAZITAXEL |  | € 13,794 | 569 | € 3,302 | 262 | € 2,260 | 167 | € 1,042 | 95 |
| OLAPARIB |  | € 13,354 | 272 | € 757 | 59 | € 674 | 52 | € 83 | 7 |
| POMALIDOMIDE |  | € 12,642 | 209 | € 2,171 | 96 | € 1,322 | 55 | € 849 | 41 |
| RUXOLITINIB |  | € 11,070 | 305 | € 345 | 46 | € 248 | 35 | € 96 | 11 |
| TRAMETINIB |  | € 10,591 | 329 | € 1,676 | 131 | € 1,337 | 103 | € 339 | 28 |
| DABRAFENIB |  | € 10,542 | 330 | € 1,615 | 132 | € 1,264 | 101 | € 350 | 31 |
| ENCORAFENIB |  | € 10,320 | 266 | € 1,269 | 102 | € 799 | 71 | € 469 | 31 |
| CARFILZOMIB |  | € 9,197 | 215 | € 1,503 | 96 | € 1,052 | 59 | € 450 | 37 |
| RADIUM RA-223 DICHLORIDE |  | € 8,323 | 377 | € 1,911 | 145 | € 1,307 | 99 | € 604 | 46 |
| BINIMETINIB |  | € 8,236 | 244 | € 1,017 | 92 | € 678 | 67 | € 339 | 25 |
| TRASTUZUMAB EMTANSINE |  | € 6,969 | 160 | € 628 | 47 | € 482 | 35 | € 146 | 12 |
| VENETOCLAX |  | € 6,083 | 115 | € 161 | 25 | € 123 | 15 | € 37 | 10 |
| ALECTINIB |  | € 4,579 | 85 | € 256 | 19 | € 173 | 13 | € 83 | 6 |
| DASATINIB |  | € 3,951 | 158 | € 331 | 47 | € 290 | 40 | € 41 | 7 |
| NIRAPARIB |  | € 3,800 | 120 | € 661 | 51 | € 615 | 46 | € 45 | 5 |
| NILOTINIB |  | € 2,153 | 84 | € 161 | 24 | € 161 | 24 | € 0 | 0 |
| **Totaal** |  | **€ 796,092** | **26308** | **€ 82,549** | **8084** | **€ 59,274** | **5696** | **€ 23,274** | **2388** |
|  | | | | | | | | | |
|  | | | | | | | | | |
|  | | | | | | | | | |
|  | | | | | | | | | |
| **2021** | | | | | | | | | |
| **Anti-cancer medicines** |  | **Overall costs**  **(x 1,000)** | **Overall No. Treatments** | **Costs ETD total**  **(x 1,000)** | **No.**  **Treatments ETD total** | **Costs EDT survivors**  **(x 1,000)** | **No. EDT survivors** | **Costs ETD deaths**  **(x 1,000)** | **No. ETD deaths** |
| PEMBROLIZUMAB |  | € 198,015 | 4281 | € 25,340 | 1741 | € 16,039 | 957 | € 9,300 | 784 |
| DARATUMUMAB |  | € 94,298 | 1110 | € 7,130 | 216 | € 4,853 | 133 | € 2,276 | 83 |
| NIVOLUMAB |  | € 58,632 | 1895 | € 6,032 | 791 | € 4,696 | 578 | € 1,336 | 213 |
| DURVALUMAB |  | € 52,441 | 814 | € 3,502 | 206 | € 3,219 | 190 | € 282 | 16 |
| LENALIDOMIDE |  | € 43,265 | 1337 | € 3,686 | 350 | € 2,714 | 253 | € 972 | 97 |
| OSIMERTINIB |  | € 32,483 | 501 | € 1,436 | 93 | € 562 | 36 | € 873 | 57 |
| ENZALUTAMIDE |  | € 32,401 | 1377 | € 2,410 | 333 | € 1,869 | 241 | € 540 | 92 |
| ABIRATERON |  | € 31,379 | 1323 | € 2,711 | 361 | € 1,978 | 246 | € 732 | 115 |
| PERTUZUMAB |  | € 29,408 | 1368 | € 2,798 | 261 | € 2,653 | 242 | € 145 | 19 |
| IBRUTINIB |  | € 22,527 | 430 | € 1,407 | 106 | € 868 | 65 | € 538 | 41 |
| PALBOCICLIB |  | € 21,263 | 1220 | € 1,666 | 319 | € 1,373 | 256 | € 292 | 63 |
| TRASTUZUMAB EMTANSINE |  | € 20,043 | 500 | € 1,915 | 135 | € 1,740 | 117 | € 175 | 18 |
| BEVACIZUMAB |  | € 19,224 | 2444 | € 2,927 | 924 | € 2,351 | 715 | € 576 | 209 |
| TRASTUZUMAB |  | € 17,421 | 2215 | € 346 | 178 | € 246 | 120 | € 100 | 58 |
| POMALIDOMIDE |  | € 16,819 | 300 | € 2,402 | 126 | € 1,408 | 68 | € 993 | 58 |
| TRAMETINIB |  | € 14,873 | 429 | € 1,949 | 169 | € 1,327 | 123 | € 621 | 46 |
| RITUXIMAB |  | € 14,728 | 3808 | € 2,274 | 1143 | € 1,908 | 888 | € 365 | 255 |
| DABRAFENIB |  | € 14,645 | 428 | € 1,776 | 158 | € 1,221 | 114 | € 554 | 44 |
| OLAPARIB |  | € 14,419 | 329 | € 1,076 | 81 | € 989 | 74 | € 86 | 7 |
| ENCORAFENIB |  | € 11,668 | 363 | € 1,522 | 142 | € 993 | 84 | € 528 | 58 |
| RUXOLITINIB |  | € 11,411 | 324 | € 356 | 50 | € 237 | 34 | € 119 | 16 |
| CABAZITAXEL |  | € 10,474 | 500 | € 2,934 | 244 | € 2,209 | 175 | € 725 | 69 |
| RADIUM RA-223 DICHLORIDE |  | € 9,351 | 418 | € 2,015 | 154 | € 1,365 | 102 | € 649 | 52 |
| CARFILZOMIB |  | € 8,853 | 206 | € 1,405 | 92 | € 931 | 58 | € 474 | 34 |
| NIRAPARIB |  | € 7,933 | 227 | € 1,221 | 88 | € 1,187 | 83 | € 34 | 5 |
| BINIMETINIB |  | € 7,818 | 255 | € 927 | 93 | € 627 | 56 | € 299 | 37 |
| VENETOCLAX |  | € 7,175 | 131 | € 75 | 16 | € 46 | 10 | € 28 | 6 |
| ALECTINIB |  | € 4,073 | 72 | € 178 | 14 | € 143 | 11 | € 34 | 3 |
| DASATINIB |  | € 4,037 | 172 | € 220 | 31 | € 202 | 28 | € 18 | 3 |
| NILOTINIB |  | € 1,143 | 58 | € 109 | 20 | € 92 | 16 | € 16 | 4 |
| **Totaal** |  | **€ 832,235** | **28835** | **€ 83,757** | **8635** | **€ 60,063** | **6073** | **€ 23,694** | **2562** |
|  | | | | | | | | | |
|  | | | | | | | | | |
|  | | | | | | | | | |
|  | | | | | | | | | |
| **2022** | | | | | | | | | |
| **Anti-cancer medicines** |  | **Overall costs**  **(x 1,000)** | **Overall No. Treatments** | **Costs ETD total**  **(x 1,000)** | **No.**  **Treatments ETD total** | **Costs EDT survivors**  **(x 1,000)** | **No. EDT survivors** | **Costs ETD deaths**  **(x 1,000)** | **No. ETD deaths** |
| PEMBROLIZUMAB |  | € 198,091 | 4460 | € 26,011 | 1812 | € 16,749 | 1035 | € 9,262 | 777 |
| DARATUMUMAB |  | € 121,503 | 1545 | € 7,335 | 256 | € 5,334 | 163 | € 2,001 | 93 |
| NIVOLUMAB |  | € 73,893 | 2464 | € 7,906 | 1007 | € 6,018 | 730 | € 1,887 | 277 |
| DURVALUMAB |  | € 50,487 | 805 | € 3,871 | 228 | € 3,534 | 201 | € 336 | 27 |
| OSIMERTINIB |  | € 32,476 | 499 | € 1,229 | 82 | € 702 | 37 | € 527 | 45 |
| ENZALUTAMIDE |  | € 29,729 | 1267 | € 2,114 | 293 | € 1,598 | 212 | € 515 | 81 |
| PERTUZUMAB |  | € 21,572 | 1237 | € 1,726 | 224 | € 1,607 | 207 | € 119 | 17 |
| IBRUTINIB |  | € 20,707 | 411 | € 1,551 | 103 | € 932 | 60 | € 619 | 43 |
| TRASTUZUMAB EMTANSINE |  | € 18,362 | 521 | € 1,534 | 138 | € 1,390 | 120 | € 144 | 18 |
| ABIRATERON |  | € 17,639 | 1764 | € 2,106 | 427 | € 1,637 | 316 | € 468 | 111 |
| PALBOCICLIB |  | € 17,340 | 1003 | € 1,204 | 247 | € 1,008 | 196 | € 196 | 51 |
| POMALIDOMIDE |  | € 15,134 | 279 | € 1,642 | 103 | € 1,109 | 61 | € 533 | 42 |
| TRAMETINIB |  | € 14,331 | 388 | € 1,685 | 144 | € 1,178 | 102 | € 507 | 42 |
| DABRAFENIB |  | € 14,019 | 394 | € 1,763 | 148 | € 1,243 | 106 | € 520 | 42 |
| TRASTUZUMAB |  | € 12,511 | 2135 | € 207 | 156 | € 142 | 96 | € 65 | 60 |
| ENCORAFENIB |  | € 12,432 | 385 | € 1,652 | 143 | € 956 | 79 | € 696 | 64 |
| BEVACIZUMAB |  | € 11,803 | 2332 | € 1,660 | 889 | € 1,345 | 696 | € 315 | 193 |
| RITUXIMAB |  | € 10,966 | 3784 | € 1,734 | 1135 | € 1,524 | 927 | € 209 | 208 |
| RUXOLITINIB |  | € 10,930 | 323 | € 393 | 52 | € 285 | 37 | € 108 | 15 |
| VENETOCLAX |  | € 10,429 | 383 | € 758 | 153 | € 465 | 95 | € 293 | 58 |
| CARFILZOMIB |  | € 10,280 | 227 | € 1,278 | 90 | € 858 | 52 | € 419 | 38 |
| OLAPARIB |  | € 10,162 | 241 | € 673 | 61 | € 623 | 55 | € 49 | 6 |
| NIRAPARIB |  | € 9,328 | 251 | € 1,181 | 84 | € 1,165 | 83 | € 16 | 1 |
| CABAZITAXEL |  | € 8,768 | 601 | € 2,177 | 287 | € 1,590 | 192 | € 586 | 95 |
| BINIMETINIB |  | € 8,615 | 265 | € 1,001 | 88 | € 580 | 49 | € 420 | 39 |
| RADIUM RA-223 DICHLORIDE |  | € 7,643 | 366 | € 1,762 | 150 | € 1,331 | 109 | € 430 | 41 |
| ALECTINIB |  | € 4,690 | 93 | € 245 | 19 | € 150 | 12 | € 94 | 7 |
| LENALIDOMIDE |  | € 4,562 | 1674 | € 735 | 453 | € 548 | 311 | € 186 | 142 |
| DASATINIB |  | € 2,615 | 179 | € 206 | 39 | € 173 | 31 | € 33 | 8 |
| NILOTINIB |  | € 1,695 | 67 | € 98 | 14 | € 98 | 14 | € 0 | 0 |
| **Totaal** |  | € 782,729 | 30343 | € 77,452 | 9025 | € 55,885 | 6384 | € 21,567 | 2641 |

**Supplementary table B:** Overview of all parameters included in this analysis per year.
