# Supplementary material for: Cost implications of early treatment discontinuation in cancer: a real-world data analysis
Source: Oncologist. 2026 Feb 25;31(4):oyaf436. doi: 10.1093/oncolo/oyaf436 (PMC12995430; doi:10.1093/oncolo/oyaf436)
Supplement: oyaf436_Supplementary_Data [file oyaf436_supplementary_data.zip › 20251110 Supplementary formulas.docx]

**Supplementary materials**

**Formula’s***.*

$$C_{medicine}= C_{indication x} +C_{indication y}+\ldots$$

*Formula 1 Sum of costs of all oncological and malignant hematological indication code per medicine. The included costs are adjusted to the subgroup calculated. Overall included all costs, ETD included only treatments <3 months. C_medicine_ = cost of an anticancer medicine.*

$$C_{total}={C_{medicine 1}+C}_{medicine 2}+ C_{medicine 3}+ .....$$

*Formula 2 Sum of all medications within a year. The costs per medicine depend on subgroup, which influences which costs are included in the C_medicine_ calculation. The same formula is used to calculate the total ETD costs and ETD costs per subgroup. C_total_ETD_: total costs of all included medicines.*
